# Digital Cognitive Rehabilitation Platforms for Older Adults in Portugal: A Systematic Review

**DOI:** 10.3390/ijerph23040453

**Published:** 2026-04-01

**Authors:** Ana Raposo, Fabiana Gonçalves, Levi Leonido, Liliana Mendes

**Affiliations:** 1 MUNDIS—Civic Association for Training and Culture, 5000-033 Vila Real, Portugal; anaraposo98@gmail.com (A.R.); fabianafsg4@outlook.pt (F.G.); 2 Catholic University of Portugal, Center for Research in Science and Technology of the Arts, 4169-005 Porto, Portugal; levileon@utad.pt; 3 University of Trás-os-Montes and Alto Douro, 5000-801 Vila Real, Portugal; 4 School of Psychology and Life Sciences, Universidade Lusófona (EPCV), 1749-024 Lisbon, Portugal; 5 Coimbra Institute for Biomedical Imaging and Translational Research, University of Coimbra, 3000-548 Coimbra, Portugal; 6 Institute for Nuclear Sciences Applied to Health (CIBIT/ICNAS), University of Coimbra, 3000-548 Coimbra, Portugal

**Keywords:** cognitive telerehabilitation, e-therapy, elderly, mild cognitive impairment, Alzheimer’s disease, Portugal

## Abstract

**Highlights:**

**Public health relevance—How does this work relate to a public health issue?**

- With dementia cases expected to double by 2080, this work focuses on the urgent need for interventions in the prodromal stage (mild cognitive impairment), where the preservation of functional autonomy is still possible.
- This study indicates how information and communication technologies can ensure continuity of care, especially for an elderly population that faces barriers to digital literacy and limited access to traditional clinicians.

**Public health significance—Why is this work of significance to public health?**

- The review highlights that digital platforms, especially those using non-immersive virtual reality, are ecologically valid tools, offering a more realistic assessment and training than traditional paper-and-pencil tests.
- This study represents a shift in rehabilitation philosophy, concluding that multimodal interventions combining physical and cognitive training significantly improve outcomes.

**Public health implications—What are the key implications or messages for practitioners, policy makers and/or researchers in public health?**

- The results suggest that investing in web-based and telerehabilitation platforms can reduce the burden on healthcare institutions, as these tools allow a single clinician to monitor multiple users remotely.
- Digital literacy is not an insurmountable barrier if the tools are culturally adapted (e.g., using Portuguese proverbs) and involve the end user in the design process. This ensures long-term adherence and reduces “institutional mistrusts”.

**Abstract:**

Portugal’s demographic ageing calls for effective strategies to address mild cognitive impairment (MCI) and Alzheimer’s Disease (AD). However, fragmented evidence on digital tools limits their clinical application. This review aimed to map the landscape of validated digital cognitive rehabilitation platforms in Portugal for older adults with MCI and AD and to analyze their effectiveness, usability, and implementation barriers. Following PRISMA 2020 guidelines, seven studies published between 2015 and 2025 were identified from PubMed, Scopus, and ScienceDirect, complemented by manual searches and platform website analysis. Methodological quality, assessed using the Joanna Briggs Institute (JBI) tools, ranged from 69% to 100%. The included studies evaluated platforms such as the Systemic Lisbon Battery (SLB), Digi&Mind, NeuroVRehab.PT, and the Fit4Alz project. Findings indicate improvements in global cognition, executive functioning, and attention. Multimodal interventions combining digital cognitive training and physical exercise produced more consistent cognitive benefits than isolated approaches. Despite initially low digital literacy among older adults, high adherence and motivation were reported, supported by gamification, user-centred design, and cultural adaptation. Although Portuguese digital platforms show strong potential for cognitive rehabilitation, the evidence base is constrained by methodological heterogeneity, small sample sizes, and short intervention durations. Future research should prioritize long-term follow-up and remote monitoring through telerehabilitation.

## 1. Introduction

Increasing life expectancy has led to a higher prevalence of age-related disorders. In Portugal, the number of dementia cases is projected to double by 2080, with prevalence estimates ranging from 2.1% and 5.0% [1]. In this context, effective management of dementia, characterized by a significant cognitive decline, as well as prodromal conditions such as mild cognitive impairment (MCI), is essential. MCI involves deterioration in cognitive domains such as attention, memory, or perceptual–motor functions, while functional autonomy is largely preserved, and daily activities remain relatively unaffected, unlike in more advanced stages such as Alzheimer’s Disease (AD) [2].

To slow cognitive decline, empirical evidence highlights participation in cognitively stimulating activities as a protective factor [3] promoting neuroplasticity and strengthening cognitive reserve [4,5]. The literature distinguishes three main intervention modalities for individuals with MCI: (i) cognitive training, which involves structured and repetitive practice of specific cognitive domains, using computerized or conventional methods to improve deficits and monitor performance, with the aim of preserving or enhancing functional abilities [6]; (ii) cognitive stimulation, which emphasizes social engagement and global mental functioning, typically delivered in group contexts; and (iii) cognitive rehabilitation, an individualized and compensatory approach focused on functional goals related to daily living and on maintaining autonomy and quality of life.

Traditional paper-and-pencil cognitive interventions continue to be widely used in rehabilitation settings due to their accessibility and established clinical validity [7]. However, advances in information and communication technologies (ICT) have facilitated a transition to web-based platforms, telerehabilitation and virtual reality (VR) systems [8,9]. This growth is also evident in the Portuguese context, where progress in e-health currently ranks above the European Union average [10].

In the psychology field, these computerized or digital approaches, often referred to as internet-delivered interventions, e-therapy or web-based interventions, are characterized by the implementation of structured therapeutic protocols and may incorporate gamification strategies or serious games, with the aim of increasing therapeutic adherence and patient engagement [11,12,13]. Compared to the traditional format, digital modalities offer greater flexibility for patients, as well as automated monitoring and adaptive tools for clinicians [14].

Although evidence suggests that digital interventions are effective in mitigating cognitive frailty and promoting continuity of care in the home setting [15], their applicability among older adults remains limited due to barriers such as restricted internet access, low digital literacy, and physical or cognitive impairments that hinder the effective use of digital interfaces [16,17].

The situation in Portugal reflects these difficulties through marked demographic and geographical disparities. Data from the TRIO report indicate that only 60% of the population aged between 55 and 74 have internet access, a figure significantly lower than the national average of 83% [18]. Furthermore, there is a critical regional disparity: whilst in 2025, in Portugal, 65.5% of residents in urban areas had a level of digital literacy equal to or above the basic level, in rural areas of the country this percentage stood at 49.3% [19]. This divide not only limits the reach of technology-based interventions but also exacerbates the vulnerability of older people living in peripheral areas, subjecting them to a condition of dual isolation: physical and digital.

This situation creates a paradox: while studies indicate that older adults may demonstrate high adherence when interventions are designed with strong usability principles, limited institutional trust among healthcare professionals, as well as limited access to these platforms, may constrain the integration of these solutions into routine clinical practice [20,21].

To address this gap, it is essential to identify digital solutions that can be effectively integrated into routine care. In Portugal, however, the fragmentation of information regarding cognitive rehabilitation platforms hampers their real-world implementation and limits the identification of gaps that could guide the development of new tools. Consequently, only a limited number of digital platforms are currently adapted to the Portuguese context.

This limitation is reflected in the national scientific evidence. Recent systematic reviews have not specifically focused on digital cognitive rehabilitation platforms developed in Portugal. Instead, the most recent articles have addressed other populations [22] or alternative rehabilitation approaches, such as VR [23] or brain stimulation [24]. Even when digital platforms are considered, the emphasis often lies on gamification features and game mechanics, both in clinical populations [25] and in elderly care settings [26], while structured and validated tools tailored to national clinical practice remain underexplored. Therefore, there is a need to examine the methodological rigour and clinical applicability of solutions adapted to the Portuguese context to address this gap.

Against this background, this systematic review aims to map the landscape of digital cognitive rehabilitation platforms in Portugal for older adults (>65 years) across the cognitive decline continuum, from subjective memory complaints and MCI to AD, identifying validated tools. Specifically, this study examines the contexts of application and effect of digital interventions on cognitive and functional outcomes and explores whether results vary according to intervention format. In addition, it seeks to identify key barriers to implementation, such as digital literacy and sustainability, with a primary focus on tools developed in Portuguese.

## 2. Materials and Methods

This systematic review was conducted in accordance with the PRISMA 2020 guidelines (Preferred Reporting Items for Systematic Reviews and Meta-Analyses), (Supplementary Materials). The research question and operational framework were structured using the PICO-TL approach (Population, Intervention, Comparison, Outcomes, Time, and Location) [27].

### 2.1. Eligibility Criteria

Eligibility criteria were defined to identify studies addressing validated digital cognitive rehabilitation interventions for the Portuguese population. Studies were included if they: (a) involved participants aged ≥ 65 years, including healthy older adults and individuals with subjective memory complaints, MCI, or AD; (b) examined interventions based on digital platforms, applications, or software dedicated to cognitive rehabilitation and validated for the Portuguese population; (c) potentially incorporated multimodal components; (d) reported the processes of design, development, adaptation and/or technical validation of the software or digital platform; (e) provided empirical data on intervention effectiveness at cognitive, functional, or behavioural levels; (f) were written in Portuguese or English; and (g) employed quantitative, qualitative, or and mixed-methods designs.

Studies were excluded if: (i) participants had a primary severe psychiatric condition; (ii) interventions were exclusively traditional or based solely on transcranial stimulation; (iii) technologies relied exclusively on immersive VR; or (iv) they were non-empirical studies, synthesis papers (systematic reviews or meta-analyses), non-interventional observational studies, or grey literature, including conference abstracts, books, book chapters, dissertations, commentaries, study protocols, or case reports.

### 2.2. Research Strategy and Sources of Information

The bibliographic search was conducted on 2 December 2025, using the PubMed, Scopus, and ScienceDirect databases. Additional databases (Apa PyscNet and SciELO) were consulted; however, no eligible records were identified. The search was restricted to articles published between January 2015 and December 2025 in Portuguese or English. The full electronic search strategies for each database are provided in Appendix A, in accordance with PRISMA 2020 recommendations.

In addition, a complementary manual search of the reference lists of included studies was conducted on 23 December 2025 to identify potentially relevant publications not captured in the initial search. Given the geographical specificity of the review and the limited number of indexed studies focusing on Portugal, a further targeted search was conducted on 29 December 2025. This involved consulting the official website of the Systemic Lisbon Battery (SLB) and reviewing associated technical and scientific publications referenced therein.

**Figure 1:**
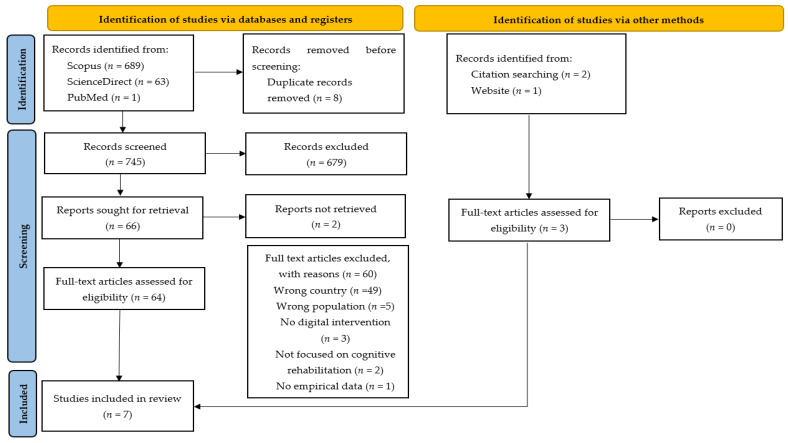
PRISMA 2020 flow diagram for systematic reviews, which includes searches of databases, registers, and other sources. From Page, MJ, McKenzie, JE, Bossuyt, PM, Boutron, I., Hoffmann, TC, Mulrow, CD, Shamseer, L., Tetzlaff, JM, Akl, EA, Brennan, SE, Chou, R., Glanville, J., Grimshaw, JM, Hróbjartsson, A., Lalu, MM, Li, T., Loder, E.W., Mayo-Wilson, E., McDonald, S., Moher, D. (2021) [28]. The PRISMA 2020 statement: an updated guideline for reporting systematic reviews. *BMJ*, 372(71). https://doi.org/10.1136/bmj.n71 [28].

The entire identification and selection process was systematically documented and used to develop the PRISMA 2020 flow diagram (Figure 1).

### 2.3. Search Syntax

The search strategy was developed by combining controlled vocabulary and free-text terms using Boolean operators (AND, OR). The search structure encompassed the following domains:


**Group**

**Concept**

**Search Terms**
APopulation(“aged” OR “elderly” OR “geriatric” OR “geriatric population”)BCondition(“mild cognitive impairment” OR “memory impairment” OR “cognitive decline”)CIntervention(“cognitive rehabilitation” OR “cognitive training” OR “neuropsychological rehabilitation” OR “cognitive remediation”)DTechnology(“digital health” OR “telemedicine” OR “telerehabilitation” OR “mobile application” OR “serious game”/“serious games” OR “exergame” OR “software” OR “app” OR “computer-assisted”)ELocation(“portugal” OR “portuguese”)

### 2.4. Study Selection Process

Bibliographic reference management and initial screening were conducted using the Rayyan platform. The selection process comprised two sequential stages:

1. Initial screening: Screening and eligibility assessment were conducted independently by two reviewers. Discrepancies were resolved through discussion and, when necessary, consultation with a third reviewer. Due to limitations in data extraction, interrater agreement (e.g., Cohen’s kappa) could not be calculated. However, all records were independently screened by two reviewers, with discrepancies resolved through discussion and consultation with a third reviewer, ensuring methodological rigour and minimizing selection bias.
2. Full-text assessment: Articles retained after screening underwent independent full-text review by the same two reviewers to confirm compliance with eligibility criteria. Reasons for exclusion at this stage were systematically documented. Final inclusion was confirmed only after full agreement with the review protocol, ensuring the methodological rigour of subsequent data extraction.

### 2.5. Data Extraction

Data extraction was conducted by one reviewer using a standardized extraction form based on PRISMA recommendations [28] and specifically adapted for this review to ensure consistency and uniformity. The form captured detailed information on authorship, year of publication, study objectives, sample characteristics, intervention features (platform type, duration, and delivery mode), and key outcomes (cognitive, functional, and behavioural), as well as study conclusions.

**Figure A1:**
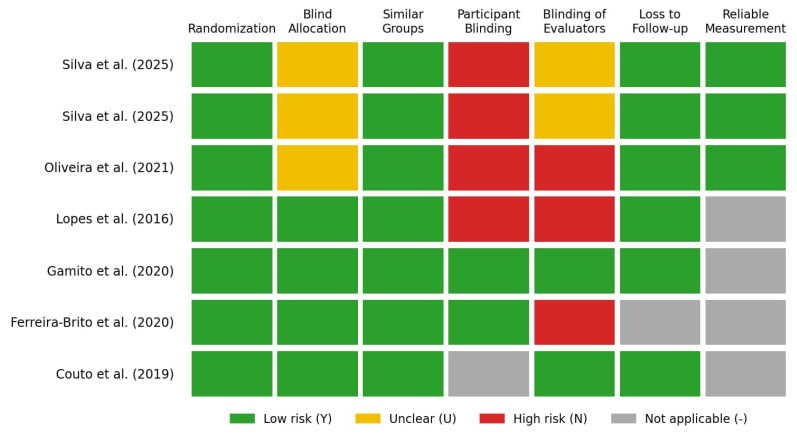
Domains of bias risk. Note. Randomization—Suitability of the randomization process (for experimental studies); Blind Allocation—Whether the allocation to the groups was blinded to participants and researchers; Similar Groups—Initial comparability of the groups before the intervention; Participant Blinding—Blinding of the participants; Blinding of Evaluators/Outcome Assessors; Loss to Follow-up—Risk of bias due to dropouts or loss to follow-up; Reliable Measurement—Validity and reliability of the measurements used. All judgments were based on the methodological information explicitly reported in the original studies [29,30,31,32,33,34,35].

To minimize the risk of bias and enhance the robustness of the evidence, methodological quality was assessed using the Joanna Briggs Institute (JBI) clinical appraisal checklists. This evaluation focused on internal validity and methodological rigour. Checklist items were subsequently aggregated into conceptual domains of risk of bias (Figure A1), in line with JBI guidance, encompassing selection, performance, detection, attrition, and reporting biases.

The database search yielded 753 records: Scopus (*n* = 689), ScienceDirect (*n* = 63), and PubMed (*n* = 1). The low number of records retrieved from PubMed is attributable to the geographical specificity of the phenomenon under study. After duplicate removal, 745 records remained for screening.

Of these, 64 articles underwent full-text review, resulting in the inclusion of four studies [29,30,31,32]. A targeted search based on a known Portuguese cognitive rehabilitation platform identified one additional study [33]. Reference list screening yielded two further eligible studies [34,35], resulting in a total of seven studies included in the review.

The primary reasons for exclusion were studies conducted outside Portugal (*n* = 49) and inappropriate target population (*n* = 5). Additional exclusions were due to the absence of a digital intervention component (*n* = 3), lack of focus on cognitive rehabilitation (*n* = 2), and absence of empirical data on intervention use (*n* = 1). These reasons are also presented in the PRISMA 2020 flow diagram (Figure 1).

### 2.6. Protocol and Registration

This systematic review was conducted according to a predefined protocol, which was retrospectively registered on the Open Science Framework (OSF; DOI: https://doi.org/10.17605/OSF.IO/ZAK2X, accessed on 14 November 2025). No methodological changes were made after registration.

## 3. Results

### 3.1. Study Selection

Figure 1 illustrates the study selection process. A total of seven empirical studies met the inclusion criteria and were included in the review. Given the substantial methodological heterogeneity across studies, findings were synthesized using a narrative approach. The included studies examined digital platforms for cognitive rehabilitation targeting older adults in Portugal. A detailed synthesis of authorship, study objectives, sample characteristics, assessment measures, intervention features, and key outcomes is presented in Table 1.

### 3.2. Characteristics of the Studies and Participants

The included studies addressed both the development and validation of digital cognitive rehabilitation platforms and the evaluation of their effectiveness and usability. Methodologically, the corpus comprised three randomized controlled trials, one controlled clinical trial, and one single-group intervention study. In addition, the review included one cross-sectional technological validation study and one mixed-methods proof-of-concept user-centred design study. Across all studies, a total of 411 participants were included, with a predominance of female participants and a mean age above 70 years. Clinical profiles ranged from healthy older adults to individuals with cognitive decline and AD.

Regarding intervention protocols, the main digital solutions identified were the SLB [30,31,33], the multidomain Digi&Mind programme [34], the NeuroVRehab.pt platform [29], and the integration of digital cognitive games into physical exercise programmes, namely, the Fit4Alz project [32,35]. The core characteristics of these platforms are summarized in Table 2, providing a comparative overview of digital tools developed and implemented in Portugal.

Interventions were delivered using diverse technological formats, including tablets, computers, and web-based platforms, and were implemented across institutional, community, and clinical settings. Effectiveness and usability were evaluated using neuropsychological instruments validated for the Portuguese population, most commonly the Montreal Cognitive Assessment (MoCA) and the Mini-Mental State Examination (MMSE) for global cognition, and the Frontal Assessment Battery (FAB) and the Trail Making Test (TMT) for executive functions.

When available, effect sizes were considered to indicate the magnitude of intervention effects. However, confidence intervals were not consistently reported, and several studies, particularly those focused on usability and technological validation, did not provide effect size estimates.

### 3.3. Risk of Bias

Two independent reviewers assessed the methodological quality and risk of bias of the included studies using the Joanna Briggs Institute (JBI) critical appraisal tools. Each JBI checklist item was independently rated based on explicit reporting in the original studies, following JBI guidance for critical appraisal. No disagreements arose during the assessment process. Overall methodological quality ranged from moderate to high, with scores varying between 69% and 100% (see Table A1, Appendix B). Detailed item-level ratings for each JBI checklist item are provided in Table A2 (Appendix B).

In general, randomized controlled trials (RCTs) demonstrated strong methodological rigour, particularly in randomization procedures and baseline group comparability. Both studies by Silva et al. [32,35] achieved quality scores of 69%, indicating a moderate overall risk of bias, with the primary limitation related to the lack of blinding and allocation concealment. The pilot trial by Oliveira et al. [31] also demonstrated moderate methodological quality (69%) with adequate measurement precision using non-immersive VR, despite limitations inherent to small-scale designs.

Among non-randomized studies, which primarily focused on technological and functional validation of digital platforms, methodological fidelity was consistently high. The study by Gamito et al. [30] achieved the maximum quality score (100%), indicating strong robustness in the evaluation of the SLB platform. Other studies [29,33,34] obtained quality scores of 78%, 75%, and 89%, respectively, reflecting rigour in cognitive assessment, usability evaluation, and user-centred design processes. Across non-randomized studies, higher risk of bias was mainly associated with the domains of randomization and allocation concealment, which is consistent with their methodological focus on technical validation and usability rather than comparative effectiveness testing.

Despite the generally moderate to high methodological quality observed across studies, several sources of bias may have influenced the reported outcomes. In particular, the lack of blinding and allocation concealment, identified in some randomized controlled trials, may have contributed to an overestimation of intervention effects, especially in outcomes related to cognitive performance, which may be susceptible to expectation and assessor-related biases. Furthermore, the predominance of non-randomized designs focused on technological validation limits the strength of causal inferences regarding intervention effectiveness. These studies, while methodologically robust in usability and feasibility assessment, are inherently more prone to selection bias and lack of comparability between groups, which may inflate perceived intervention benefits. Additionally, small sample sizes and the pilot nature of some studies reduce statistical power, increasing the likelihood of type II errors or unstable effect estimates. This is particularly relevant in studies reporting non-significant or modest improvements, where insufficient power may mask true intervention effects. Therefore, the positive effects reported across studies, particularly improvements in global cognition and executive functions, should be interpreted with caution, considering the potential influence of these methodological limitations.

### 3.4. Characterization of Digital Platforms and Application Contexts in Portugal

The results revealed four main typologies of digital platforms within the Portuguese context. The first is the SLB, an interactive platform that uses non-immersive VR scenarios to simulate daily life activities in a controlled environment [30,31,33]. The second typology comprises programmes for mobile devices and tablets, such as Digi&Mind, which focuses on multidomain cognitive stimulation [34], and NeuroVRehab.PT, which employs photorealistic virtual environments, such as a virtual supermarket, to simulate everyday tasks [29]. The third typology includes cognitive game software integrated into physical training programmes, as exemplified by the Fit4Alz project [32,35].

Regarding application contexts, interventions were implemented in both institutional and community settings, allowing for technical supervision in day-care centres and municipal gyms. Cultural adaptation emerged as a relevant feature in the study by Couto et al. [34], which incorporated culturally meaningful content, including popular Portuguese proverbs, into the intervention design. This finding is supported by Ferreira-Brito et al. [29], who adopted a user-centred design approach to ensure that virtual scenarios were culturally relevant, thereby minimizing barriers to platform adoption and use.

### 3.5. Impact of Digital Interventions on Cognition in Older Adults: Global Cognition and Specific Cognitive Domains

Results concerning global cognition showed some heterogeneity; nevertheless, an overall positive trend towards the effectiveness of functional digital platforms was observed. Significant improvements in global cognitive performance were reported following the use of the SLB in older adults with AD after 12 weeks of intervention, with sessions lasting 45 min [31], with the authors describing a large effect size, although confidence intervals were not reported. These findings are consistent with those reported by Gamito et al. [30], in which healthy older adults also demonstrated statistically significant improvements in global cognition and executive functions after only six weeks, although effect sizes were not reported.

With regard to executive functions, Oliveira et al. [31] did not observe statistically significant improvements, despite positive trends, especially in performance on the TMT. In contrast, Gamito et al. [30] reported consistent gains in attention and executive functioning in the experimental group. Similarly, Ferreira-Brito et al. [29] suggested that VR-based activities of daily living may have potential for the rehabilitation of specific cognitive domains, such as working memory and cognitive flexibility, in individuals with MCI; however, these findings should be interpreted as exploratory.

Conversely, Silva et al. [32] did not report statistically significant changes in global cognition across intervention groups of the Fit4Alz project. However, moderate effect sizes were reported in some outcomes (e.g., Cohen’s d = 0.598–0.698), suggesting that the intervention may still have had a meaningful impact, potentially limited by factors such as intervention duration or measurement sensitivity. The authors suggested that MoCA may not be sufficiently sensitive to detect cognitive changes over a 12-week intervention period, and that longer or more intensive interventions may be required.

### 3.6. Usability and Acceptability

Overall, the digital platforms included in this review were well accepted by the Portuguese older population. Digi&Mind [34] demonstrated good acceptability among both professionals and older adults, despite 75% of participants reporting no prior experience with tablet devices. High usability indices were also reported for the NeuroVRehab.PT platform [29], although some difficulties were identified, particularly in the interpretation of moving arrows and navigation the virtual space. Nevertheless, the use of photorealistic interfaces appeared to mitigate barriers related to limited technological familiarity.

Within the SLB platform, tasks such as Virtual Kitchen and Art Gallery tasks demonstrated convergent validity with traditional neuropsychological assessments [33]. This evidence is further supported by Gamito et al. [30], who reported improvements in well-being and life satisfaction among healthy older adults. Motivation and adherence were largely sustained by the playful nature of the interventions and the incorporation of gamification strategies, particularly when the content was culturally meaningful. These features appeared to help overcome fatigue commonly associated with traditional cognitive training approaches [30,31,32].

### 3.7. Memory and Physical Exercise

In the study by Silva et al. [35], groups combining physical activity with cognitive training showed improvements in global cognition, with increased performance on cognitive tasks related to memory and executive functions compared with the control group. Groups engaging exclusively in physical activity also demonstrated cognitive gains, although of a smaller magnitude.

In contrast, Silva et al. [32] did not observe statistically significant changes in global cognition in any intervention group. However, moderate effect sizes (e.g., Cohen’s d = 0.598–0.698) suggest that the intervention may have had underlying effects not detected by statistical testing, highlighting the influence of methodological factors such as intervention duration, intensity, and sensitivity of cognitive assessment instruments.

### 3.8. Ecological Validity and Functional Outcomes

The SLB stands out for its high ecological validity, as it employs virtual scenarios that replicate everyday tasks, such as the Virtual Kitchen, Pharmacy, and Wardrobe Management tasks. These environments enable cognitive assessment and training that closely approximate real-life functioning in older adults [33]. This approach has been associated with improvements in functional capacity and autonomy related to activities of daily living (ADLs), as well as enhanced detection of sequencing and planning errors [31,33]. Similarly, the NeuroVRehab.PT platform reinforces this ecological paradigm using a highly realistic virtual supermarket designed to stimulate executive functions, attention, memory, and calculation skills. The functional nature of these tasks suggests a greater potential for the transfer of trained cognitive skills to everyday contexts [29].

Beyond cognitive and functional outcomes, older adults reported increased confidence in their abilities, which may contribute to reducing social isolation and greater independence in daily activities [32,34]. This psychosocial impact is further supported by Gamito et al. [30], whose findings indicate statistically significant improvements in well-being and life satisfaction. Collectively, these results suggest that digital platforms may contribute to active ageing by supporting cognitive functioning, self-efficacy, and perceived quality of life.

Furthermore, multimodal interventions, such as the Fit4Alz programme, were associated with improvements in agility and dynamic balance, indicating that digital cognitive training, when integrated with physical activity, may contribute to greater functional safety and stability in older adults [32].

### 3.9. Comparative Effectiveness: Multimodal vs. Unimodal Interventions

Evidence suggests that multimodal training may enhance outcomes when compared with unimodal approaches. Exclusively digital, unimodal cognitive programmes, such as Digi&Mind, were associated with improvements in specific cognitive domains, including attention, working memory, and executive functions [34]. In contrast, multimodal programmes, such as Fit4Alz, demonstrated combined benefits for cognitive functioning and physical fitness in older adults with cognitive decline [32]. These findings are further supported by Silva et al. [35], who reported moderate effect sizes in cognitive and physical outcomes (e.g., partial η^2^ = 0.622), suggesting that combined cognitive–physical training may enhance the overall impact of interventions.

However, findings from Gamito et al. [30] indicate that unimodal VR-based interventions may approach the effectiveness of multimodal programmes and demonstrate superior efficacy compared with traditional unimodal approaches when tasks are complex and engage multiple cognitive domains. Similar conclusions were reported by Ferreira-Brito et al. [29]. Overall, these findings suggest that non-immersive VR interventions represent a robust unimodal alternative for strengthening cognitive reserve.

Overall, the interpretation of effectiveness outcomes is limited by the inconsistent reporting of effect sizes and the absence of confidence intervals across several studies, which constrains the comparability and quantitative interpretation of findings.

## 4. Discussion

The present systematic review aimed to map and critically evaluate the landscape of digital cognitive rehabilitation platforms developed and validated in Portugal for older adults across the continuum of cognitive decline. Overall, the findings support the growing role of digital health interventions as viable tools for enhancing cognitive functions, promoting autonomy and supporting active ageing. The potential effectiveness of these tools aligns with international evidence that digital health can significantly improve global cognitive function and reduce depressive symptoms in both healthy older adults and those with MCI [36]. By examining stages ranging from subjective memory complaints to AD, this review identified not only validated tools and their clinical effectiveness but also key barriers influencing real-world implementation, including digital literacy and long-term intervention sustainability. However, the strength of the available evidence remains constrained by methodological heterogeneity and limited large-scale validation.

**Paradigm shift: Ecological validity and autonomy**

The findings of this review highlight a clear paradigm shift from traditional, decontextualized cognitive rehabilitation approaches toward digitally mediated interventions characterized by high ecological validity and user-centred design. Across the included studies, older adults consistently demonstrated strong receptiveness, alongside cognitive and psychosocial benefits, particularly when interventions were tailored to usability principles and real-world relevance, as observed in programmes such as Digi&Mind [34] and NeuroVRehab.PT [29]. Notably, the latter actively involved older adults in the design and validation process, thereby addressing the so-called digital exclusion paradox and enhancing both accessibility and engagement [20]. Beyond usability, these platforms appear to engage multiple cognitive domains, such as working memory, attention and executive functioning, within functionally meaningful contexts. This integrated stimulation may support mechanisms related to cognitive reserve, moving beyond isolated domain training toward more holistic cognitive engagement [5].

A key theme emerging from this review is the shift from traditional cognitive rehabilitation formats towards digital approaches. Whilst conventional pen-and-paper methods remain valid and effective [7], studies using the SLB [30,31,33] highlight ecological validity as a major advantage of ICT-based interventions. In contrast to traditional paper-and-pencil methods, which often rely on abstract and decontextualized exercises, these ecologically valid environments enable a closer alignment between training processes and real-life functional demands. The alignment is clinically significant. By strengthening the transferability of cognitive gains to activities of daily living [33], digital interventions may contribute to the preservation of autonomy and functional independence. Given that the transition from MCI to established dementia is largely defined by loss of independence, the incorporation of ecologically valid tasks represents not only a methodological advancement but also a meaningful shift toward function-oriented rehabilitation [2].

**Intervention modalities and multimodal synergies**

With regard to intervention modalities, the results of the Fit4Alz programme [35] and non-immersive VR interventions [30] further clarify the distinctions between cognitive training, cognitive stimulation and cognitive rehabilitation. The evidence suggests that multimodal interventions, which combine digital cognitive training with physical exercise, produce more consistent and synergistic results. This phenomenon can be explained by greater recruitment of neuroplasticity mechanisms and cognitive reserve [4].

International studies support this view, indicating that bimodal or trimodal protocols (involving nutrition, psychological and social intervention alongside physical exercise and cognitive training) tend to outperform single-modality interventions in improving overall cognition, attention and executive functions. However, it is important to note that the specific combination of physical and cognitive training, as in the Fit4Alz study [35], presents mixed results in the literature [37]. This further ambiguity is exemplified by Silva et al. [32], who reported no statistically significant changes in global cognition when measured via MoCA, yet identified moderate effects in specific domains.

These findings suggest that clinical efficacy does not stem solely from the accumulation of multiple modalities. Instead, for measurable cognitive gains to occur, factors such as task complexity, intervention intensity, progression and methodological rigour are crucial. In the Portuguese context, these findings highlight that the effectiveness of digital cognitive rehabilitation depends not only on the software design but also on its integration into structured active ageing and rehabilitation programmes.

Concerning non-immersive VR, the literature highlights significant practical advantages, such as ease of administration and versatility in clinical settings, positioning it as a viable alternative to meet patient needs. In terms of clinical efficacy, recent meta-analyses [38] demonstrate significant improvements in key indicators such as global cognition (MMSE), with an effect size of 0.76, and in executive function (TMT-A), with an SMD of −3.35. These data align with those reported by Oliveira et al. [31], who also identified benefits in the MMSE (SMD = 0.17) and in the TMT-A (SMD = −1.77). Although the figures reported by Oliveira et al. [31] show a more conservative effect size than the meta-analysis cited above, both studies agree on the validation of non-immersive VR as an effective tool for cognitive enhancement.

**Barriers to digital literacy and motivational factors**

Contrary to persistent assumptions in the literature, digital literacy did not emerge as an insurmountable barrier to technology use among older adults [16]. Instead, the findings suggest that it functions as a modifiable factor, which can be mitigated through appropriate design strategies. The high levels of adherence observed in programmes such as Digi&Mind and NeuroVRehab.PT [29,34] indicate that even individuals with limited education and minimal prior exposure to technology can engage effectively when interventions incorporate gamification and user-centred design principles. These features appear to reduce initial resistance and may help mitigate the lack of institutional trust previously described in the literature [21]. In addition to design-related factors, external elements such as social support (family, friends and community), structured assistance during sessions (e.g., reminders and guidance), and the perception of technology as beneficial to health have also been identified as important facilitators of adherence [39].

Evidence from comparable contexts further supports this interpretation. In a study conducted in Spain, 62% of older adults reported curiosity about learning to use tablets; however, 43% of those with no prior experience expressed insecurity and fear of making mistakes, such as “breaking” the device or using it incorrectly [40]. These findings highlight that initial apprehension is common, but not necessarily a barrier to engagement when appropriate support is provided.

Taken together, these results suggest that digital literacy should not be conceptualized as a fixed limitation, but rather as a dynamic and context-dependent factor. Digital tools that prioritize functional relevance and cultural appropriateness may therefore play a key role in addressing the current shortage of platforms adapted to the Portuguese context. In this sense, engagement with technology may evolve from an initial barrier into a source of motivation, social interaction and emotional support, particularly in managing the challenges associated with technology use in later life [29].

**Sustainability, accessibility and the geographical challenge**

An additional challenge highlighted by this review concerns the sustainability and continuity of digital interventions. Ensuring that rehabilitation programmes do not place an excessive burden on healthcare institutions or families, while still promoting well-being and life satisfaction, remains a critical concern [30]. Evidence indicates that discontinuity of care may accelerate functional decline and increase the risk of early institutionalization [15]. From a clinical perspective, these findings underscore the importance of selecting tools that are both semantically and culturally adapted to the Portuguese context in order to support long-term engagement and adherence.

Web-based platforms and telerehabilitation are emerging as promising strategies to enhance both accessibility and sustainability within the healthcare system. The effectiveness demonstrated by interventions such as that reported by Couto et al. [34] suggests that digital technologies can support the delivery of structured cognitive rehabilitation beyond traditional clinical settings. By facilitating remote monitoring, these approaches help overcome logistical barriers, including transportation costs and physical limitations that restrict access to in-person care, while also reducing the burden on caregivers [39]. In addition, these models enable remote technical supervision, optimize resource allocation and allow a single clinician to monitor multiple users simultaneously. However, their successful implementation remains dependent on adequate infrastructure, digital support and sustained user engagement, particularly in geographically dispersed or underserved regions.

**The Portuguese context and digital exclusion**

Although Portugal shares demographic ageing trends with other European countries, the findings of this review highlight specific structural and socio-digital characteristics that justify a context-specific analysis. Compared with other EU countries, Portugal presents pronounced regional disparities in digital literacy and internet access, particularly affecting rural and ageing populations. Overcoming these barriers is particularly important in Portugal, where a significant proportion of the elderly population lives in rural or isolated areas [36]. The marked discrepancy in internet access between rural and urban areas in Portugal reinforces the need for platforms to provide functional offline versions. However, specific details regarding connectivity requirements are scarce in the literature. In this review, only the study by Ferreira-Brito et al. [29] explicitly refers to the online format, whilst the remaining authors do not specify the conditions of access.

This gap is corroborated by research indicating that most programmes require a constant internet connection [39]. Dependence on a stable network therefore remains a barrier to the democratization of access in areas with inadequate infrastructure. As an alternative to mitigate this exclusion, the use of community resources, such as public libraries or community centres, is suggested. These spaces can function as strategic bridges, ensuring the necessary connectivity within a socially integrated environment [39].

**The mediating role of the clinician**

The evidence analyzed in this review highlights the role of the clinician as an indispensable source of support who acts to promote the autonomy of older adults by offering the technical and emotional support needed to reduce anxiety, such as the “fear of technical error”, and to foster motivation with the task [34]. As corroborated by Ferreira-Brito et al. [29], this presence is particularly vital in the initial sessions to ensure the success of the intervention, focusing on guidance during the learning of the platform and on managing feelings of frustration or failure in the face of error.

In line with these findings, the external literature emphasizes that the clinician must assume a crucial mediating role, ensuring that technology functions as a facilitator rather than a barrier to the therapeutic process; digital tools are support instruments and never substitutes for the professional, as their presence is essential for monitoring emotional, psychosocial and behavioural dimensions that the software, on its own, cannot capture. Ultimately, the strength of the therapeutic alliance remains one of the strongest predictors of treatment adherence and effectiveness, reaffirming that technological innovation must always be mediated by human contact to achieve its full clinical potential.

### Limitations

While reinforcing the transformative potential of technology in cognitive rehabilitation in Portugal, these findings must be interpreted considering several methodological limitations. First, the inclusion of studies identified through manual searches may have introduced selection bias; however, this approach was necessary to capture the national landscape, given the limited indexing of Portuguese studies in major international databases.

Substantial methodological heterogeneity was observed across the included studies, including variation in technological platforms, intervention duration, and assessment instruments. In addition, small sample sizes in several studies limited statistical power and constrained the generalizability of findings to the broader Portuguese population of older adults with MCI and AD.

An additional limitation relates to the inconsistent reporting of statistical indicators across the included studies. In particular, effect sizes and 95% confidence intervals were not systematically reported, especially in studies focused on usability and technological validation. This limitation constrains the interpretation of the precision and clinical relevance of the observed effects and reduces the comparability of findings across studies.

Another important limitation concerns the scarcity of follow-up data, which precludes evaluation of the long-term sustainability of intervention effects after digital training concludes. Moreover, most interventions were delivered in controlled settings with professional mediation, making it difficult to disentangle the specific effects of the digital tools from those of therapist support and social interaction.

Furthermore, the inclusion of non-randomized and proof-of-concept studies alongside RCTs, while expanding the evidence base, limits the strength of conclusions regarding intervention effectiveness. These studies often have smaller sample sizes, lack control groups, or present higher risk of bias; therefore, observed effects should be interpreted as preliminary indications rather than definitive evidence. Future research should prioritize rigorously designed RCTs to confirm these findings.

Finally, although the search strategy included the term “VR,” this review focused exclusively on non-immersive VR interventions. This methodological decision limits the generalization of findings to immersive VR modalities but enables a more coherent analysis of interventions that are technologically accessible and widely applicable within the Portuguese context.

Future research should prioritize multicenter studies with larger and more representative samples, as well as the inclusion of maintenance and follow-up protocols to evaluate real-world functional outcomes over time. The integration of objective biological measures, such as neuroimaging and electroencephalography (EEG), would further strengthen the evidence base.

## 5. Conclusions

This systematic review characterizes the current landscape of digital cognitive rehabilitation in Portugal, highlighting a context of increasing innovation and adaptation to the specific needs of the ageing population. Overall, the findings suggest that the transition from traditional paper-and-pencil approaches to digital tools is feasible and may be associated with improvements in cognition, mood, and everyday functioning among older adults with MCI and AD.

A key finding concerns the attenuation of low digital literacy as a barrier to implementation. The included studies indicate that digital tools developed with a strong emphasis on usability and user-centred design can achieve satisfactory levels of adherence and motivation, even among older adults with limited educational attainment. Furthermore, multimodal interventions combining physical exercise with cognitive training appear to yield more consistent outcomes, although their effectiveness depends on methodological factors such as intervention intensity, task complexity and duration.

Taken together, these findings support the role of digital technologies as viable strategies for promoting active ageing, maintaining autonomy, and enhancing quality of life in older adults. Despite methodological constraints and the need for longitudinal research, investment in digital platforms validated for the Portuguese context represents a critical step toward strengthening healthcare sustainability and improving the well-being of the ageing population.

## Figures and Tables

**Table 1:** Characteristics of included studies.

Authors, Year	Main Goal	Sample		Measures	Intervention			Results
		Size	Features		Platform	Intervention Time	Application Context	
Couto et al., 2019 [34]	To describe the development and validation process of the Digi&Mind programme.	*N* = 11 (7 professionals and 4 patients).	Professionals: nurses, occupational therapists, and psychologists. Patients: average age 72, cognitive decline, 75% women.	MoCA; PSSUQ Content validation questionnaires for experts (based on relevance and clarity). Direct observation and usability assessment grids for older adults.	Web (Mind & Gait): Monitoring professionals/caregivers. Mobile (Digi & Mind): Professional-Elderly Use; Thematic rehabilitation with 2 levels adjustable by the facilitator.	9 sessions (8 individual + 1 group); Session: 45 to 60 min.	Community context.	Considered useful and interesting; quantitative and interview feedback identified areas for improvement, including error handling (web) and usability for older adults (mobile). Effect size: NR 95% CI: NR
Ferreira-Brito et al., 2020 [29]	To describe the design and characterize the perspectives on the rehabilitation potential of the NeuroVRehab.PT platform (virtual supermarket).	Phase 1: *N* = 110; Phase 2: *N* = 7.	Phase 1: 61–86 years old; 77% women; autonomous seniors responsible for their own grocery shopping. Phase 2: Healthcare professionals: psychologists, neuropsychologists, and neurologists.	Questionnaire on Shopping Behaviours (11 questions). K-MMSE-2 and K-MoCA. Star system (performance); Qualitative feedback.	Web-based (optimized for Tablet). Non-immersive VR—Virtual supermarket Three Modes: Supermarket (14 levels, 3 difficulty levels), Recipes and Shopping List. Training in cognitive functions and behavioural strategies.	Interviews/Tests of approximately 60 min. Suggested sessions of 60 min.	University in Almada) and Clinical (Neurology/Psychology).	Professionals emphasized the platform’s realism and motivational potential, while identifying therapist support as essential to manage frustration and learning demands. Effect size: NR 95% CI: NR
Gamito et al., 2020 [30]	To evaluate the effectiveness of a programme of stimulation Cognitive-based VR (VR-CS), compared to a traditional paper-and-pencil programme (PP-CS), in older adults.	*N* = 43 (experimental group (*n* = 23) and control group (*n* = 20)).	67–87 years old; Fluent in Portuguese.	MoCA; FAB; WMS-R; RCF; d2 Test of Attention; GDS-15; SWLS; IADL.	SLB: Non-immersive VR with virtual urban and domestic environments, with functional tasks of daily life activities.	6 weeks Experimental group2 sessions/week of 30 min Control Group:1 session/week of 60 min	Community context	VR-based cognitive stimulation led to greater improvements in global cognition and executive functions than traditional approaches, with consistent gains in attention and selective improvements in visual memory. Effect size: NR 95% CI: NR
Lopes et al., 2016 [33]	To validate the SLB by comparing it with traditional tests.	*N* = 19.	Healthy seniors; Average age 75 years. 18 women; Low level of education (average 4.9 years).	SLB; MoCA; MMSE; FAB; RCF; TPT; IADL; WCST; GDS.	SLB: Non-immersive VR with 3 tasks—(planning, working memory, organization), VKT (selective attention, visual memory, efficiency) and AGT (attention and cognitive flexibility)	15 min for familiarization with the assessment.	Institutional context.	Performance on SLB tasks correlated with traditional neuropsychological measures, with AGT reflecting attention and cognitive flexibility and VKT reflecting attention and visual memory. Effect size: NR 95% CI: NR
Oliveira et al., 2021 [31]	To assess the feasibility of VR using the SLB programme in a cognitive stimulation format in AD.	*N* = 17 (experimental group (*n* = 10) and control group (*n* = 7)).	Average age of 83.2 years. Diagnosis of mild to moderate AD. 12 women and 5 men.	FAB; TMT A and B; MMSE; CDT; IADL; GDS-15; CDR	SLB is a computer-based version of IADL in non-immersive VR. 9 tasks (indoors: T1–T6; outdoors: T7–T9) performed on a laptop, 12 sessions with different levels of difficulty.	12 cognitive stimulation sessions, 45 min per session. 2×/week, total ≈ 9 h.	Institutional context, day centre.	Improvement in overall cognition; no significant gains in executive functions. Large effect size reported; 95% CI: NR.
Silva et al., 2025a [32]	To investigate and compare the effects of team sports versus aerobic exercise, with or without cognitive games, in reducing cognitive decline.	*N* = 50.	Average age of 69.3 ± 3.2 years. All with a MoCA score below 26. Divided into 5 groups: Team Sports (TS), TS + Cognitive, Aerobic (A), A + Cognitive and Control.	Cognitive: MoCA. Physical tests: Senior Fitness Test Battery.	Digital cognitive training: Fit4Alz, (memory, attention, and executive functions—2 games/domains; 5 difficulty levels) Web platform (PC/tablet). Combined with team sports (handball, football, basketball) or aerobic training.	Intervention programme with two 60 min sessions per week, for 12 weeks. Fit4Alz: 20 min.	Community context	Significant time-group interactions were observed for cognition, strength, and mobility, with TS and TS + C showing the greatest improvements, and TS + C demonstrating additional gains in memory and executive functioning. Moderate-to-large effect sizes observed (Cohen’s d = 0.598–0.698); 95% CI: NR
Silva et al., 2025b [35]	To evaluate the effects of aerobic and strength training, combined or not with regular cognitive training, in mitigating cognitive decline.	*N* =154.	Average age = 72.8 ± 6.1 years. 107 women and 47 men. All with a MoCA score below 26.	Cognitive: MoCA. Physical (Senior Fitness Test): Chair stand, arm curl, chair sit and reach, back scratch, foot up and go, 6 min walk or 2 min step. Anthropometric measurements: Height, body mass, and BMI.	Digital cognitive training: Fit4Alz (memory, attention and executive functions—2 games/domains; 5 difficulty levels); Aerobic and strength training programmes.	16 weeks total (12 weeks of direct intervention. Sessions of 60 min (physical) + 20 min (cognitive). 3 times a week.	Multicenter (Portugal, Serbia, Italy, and Poland) in partner facilities.	Overall physical fitness across all groups, with the aerobic plus cognitive training group showing the greatest gains in flexibility and balance. Large effect size observed (partial η^2^ = 0.622); 95% CI: NR

*Note.* When available, the effect sizes and 95% confidence intervals (CIs) are reported as presented in the original studies. Several studies did not report the effect sizes and confidence intervals. Post-Study System Usability Questionnaire—PSSUQ; Mini-Mental State Examination—MMSE; Montreal Cognitive Assessment—MoCA; Kiddie Mini-Mental State Examination—2nd Edition—K-MMSE-2; Frontal Assessment Battery—FAB; Rey Complex Figure—RCF; Toulouse Piéron Test—TPT; Wisconsin Card Sorting Test—WCST; Geriatric Depression Scale—GDS; Instrumental Activities of Daily Living Scale—IADL; Trail Making Test—TMT; Clock Drawing Test—CDT; Clinical Dementia Rating—CDR; Virtual Kitchen Task—VKT; Art Gallery Task—AGT; Team Sports—TS; Team Sports with cognitive training—TS + C; Wechsler Memory Scale–Revised—WMS-R; Geriatric Depression Scale-15—GDS-15; Satisfaction with Life Scale—SWLS; Virtual Reality-based Cognitive Stimulation—VR-CS; Paper-and-Pencil Cognitive Stimulation—PP-CS; Systemic Lisbon Battery—SLB; not reported—NR; confidence interval—CI.

**Table 2:** Comparison of digital solutions across included studies.

Author, Year	Digital Solution	Technology Type	Modality	Cognitive Domains	Support/Mediation	Customization	Gamification Motivation/Adherence
Couto et al., 2019 [34]	Digi&Mind	Mobile/Web.	Cognitive (Unimodal)	Memory, attention, executive functions.	Professional/caregiver	Two levels adjustable by the mediator.	Playful interface, themed tasks, and adjustable levels. High acceptance by the elderly.
Ferreira-Brito et al., 2020 [29]	NeuroVRehab.PT	Web-based non-immersive VR (virtual supermarket).	Cognitive (Unimodal)	Working memory, attention, executive functions, calculation.	Professional/mediator	User-centred design.	Culturally relevant photorealistic scenarios. Use of relevant challenges and daily tasks increases motivation and engagement. It reduces the digital literacy barrier.
Gamito et al., 2020 [30]	SLB	Non-immersive VR.	Cognitive (Unimodal)	Global cognition, attention, executive functions, memory.	Professional/mediator	Adaptation of the difficulty of the tasks depending on performance.	Gamified functional tasks increase motivation.
Lopes et al., 2016 [33]	SLB	Non-immersive VR.	Cognitive (Unimodal)	Attention, visual memory, executive functions.	Professional/mediator	Focus on structured tasks (VKT and AGT).	Structured tasks with immediate feedback. Positive acceptance.
Oliveira et al., 2021 [31]	SLB (computerized)	Non-immersive VR.	Cognitive (Unimodal)	Global cognition, executive functions.	Professional/mediator	Adjustable difficulty levels.	Light gamification with tasks of increasing complexity. Similarity to daily routines increases motivation.
Silva et al., 2025a [32]	Fit4Alz	Web/Tablet + physical training.	Cognitive + physical exercise (multimodal)	Memory, attention, executive functions.	Professional/mediator	5 adjustable cognitive difficulty levels.	Cognitive games integrated into physical training. -Scoring elements and difficulty levels increase motivation. High adherence in combined groups.
Silva et al., 2025b [35]	Fit4Alz	Web/Tablet + physical exercise.	Cognitive + physical exercise (multimodal)	Memory, attention, executive functions.	Professional/mediator	5 adjustable cognitive difficulty levels.	Fun digital games combined with physical exercise. Difficult adaptation maintains an appropriate challenge. High adherence despite the prolonged duration of the intervention.

*Note*. Systemic Lisbon Battery—SLB; Virtual Kitchen Task—VKT; Art Gallery Task—AGT.

## Data Availability

The original contributions presented in this study are included in the article. Further inquiries can be directed to the corresponding author.

## Supplementary Materials

The following supporting information can be downloaded at: https://www.mdpi.com/article/10.3390/ijerph23040453/s1, File S1: PRISMA 2020 Checklist.

## Abbreviation

The following abbreviations are used in this manuscript:

AD	Alzheimer’s Disease
ADLs	Activities of Daily Living
AGT	Art Gallery Task
CDR	Clinical Dementia Rating
CDT	Clock Drawing Test
FAB	Frontal Assessment Battery
GDS	Geriatric Depression Scale
IADL	Instrumental Activities of Daily Living Scale
ICTs	Information and Communication Technologies
JBI	Joanna Briggs Institute
MCI	Mild Cognitive Impairment
MMSE	Mini-Mental State Examination
MoCA	Montreal Cognitive Assessment
*n*	Subsample size
*N*	Total sample size
OSF	Open Science Framework
PRISMA	Preferred Reporting Items for Systematic Reviews and Meta-Analyses
RCF	Rey Complex Figure
RCT	Randomized Controlled Trial
SLB	Systemic Lisbon Battery
TMT	Trail Making Test
TPT	Toulouse Piéron Test
VR	Virtual Reality
VKT	Virtual Kitchen Task
WCST	Wisconsin Card Sorting Test
WMS-R	Wechsler Memory Scale–Revised

## Appendix A

This appendix presents the complete electronic search strategies used for each database (PubMed, Scopus, and ScienceDirect), including all keywords, Boolean operators, and applied filters. These strategies were developed based on the PICO-TL framework and are reported in detail to ensure transparency and reproducibility in line with PRISMA 2020 recommendations.

**Scopus**
The search was conducted using advanced search syntax, combining terms related to population, condition, intervention, technology, and location using Boolean operators (AND, OR).
Search string:
(ALL((Aged OR Elderly OR Geriatric OR “geriatric population”) AND (“Mild Cognitive Impairment” OR MCI OR “Memory Impairment” OR “Cognitive Decline”) AND (“Cognitive rehabilitation” OR “cognitive training” OR “neuropsychological rehabilitation” OR “Cognitive Remediation”) AND (“digital health” OR telemedicine OR telerehabilitation OR “mobile application” OR “serious game*” OR “virtual reality” OR exergame OR software OR app OR “computer-assisted”) AND (Portugal OR Portuguese))) AND PUBYEAR > 2014 AND PUBYEAR < 2026 AND (LIMIT-TO (DOCTYPE,”ar”)) AND (LIMIT-TO (LANGUAGE,”English”) OR LIMIT-TO (LANGUAGE,”Portuguese”))
Filters applied:
  - Publication years: 2015–2025;
  - Document type: Article;
  - Language: English and Portuguese.
Results: *n* = 689
**PubMed**
The search strategy incorporated Medical Subject Headings (MeSH) and free-text terms.
Search string:
((Aged[MeSH Terms] OR aged[Title/Abstract] OR elderly[Title/Abstract] OR geriatric[Title/Abstract] OR “geriatric population”[Title/Abstract]) AND (“Mild Cognitive Impairment”[MeSH Terms] OR “mild cognitive impairment”[Title/Abstract] OR MCI[Title/Abstract] OR “memory impairment”[Title/Abstract] OR “cognitive decline”[Title/Abstract]) AND (“Cognitive Therapy”[MeSH Terms] OR “cognitive rehabilitation”[Title/Abstract] OR “cognitive training”[Title/Abstract] OR “neuropsychological rehabilitation”[Title/Abstract] OR “cognitive remediation”[Title/Abstract]) AND (“Digital Health”[MeSH Terms] OR digital health[Title/Abstract] OR telemedicine[MeSH Terms] OR telemedicine[Title/Abstract] OR telerehabilitation[Title/Abstract] OR “mobile application”[Title/Abstract] OR “serious game”[Title/Abstract] OR “serious games”[Title/Abstract] OR “virtual reality”[MeSH Terms] OR “virtual reality”[Title/Abstract] OR exergame*[Title/Abstract] OR software[Title/Abstract] OR app[Title/Abstract] OR “computer-assisted”[Title/Abstract]) AND (Portugal[Affiliation] OR Portugal[Title/Abstract] OR Portuguese[Title/Abstract]))
Filters applied:
  - Publication years: 2015–2025.
Results: *n* = 1
Note: Additional filters (e.g., language and article type) were not applied due to database constraints but were considered during the screening phase.
**ScienceDirect**
Search string:
(Geriatric OR Elderly OR Aged) AND (“Cognitive Impairment” OR “Cognitive Decline”) AND (“Cognitive rehabilitation” OR “Cognitive training”) AND (Telerehabilitation OR “virtual reality” OR “serious game” OR “digital health”) AND (Portugal OR Portuguese)
Filters applied:
  - Publication years: 2015–2025.
Results: *n* = 63

## Appendix B

**Table A1:** Assessment of the risk of bias in included studies: JBI checklist.

Author, Year	JBI Tool	“Yes” Items	Percentage	Quality	Risk
Couto et al., 2019 [34]	Quasi-experimental	8/9	89%	High	Low
Ferreira-Brito et al., 2020 [29]	Cross-sectional	6/8	75%	High	Low
Gamito et al., 2020 [30]	Quasi-experimental	9/9	100%	High	Low
Lopes et al., 2016 [33]	Quasi-experimental	7/9	78%	High	Low
Oliveira et al., 2021 [31]	RCT	9/13	69%	Moderate	Moderate
Silva et al., 2025a [32]	RCT	9/13	69%	Moderate	Moderate
Silva et al., 2025b [35]	RCT	9/13	69%	Moderate	Moderate

Note. Methodological quality was classified based on the percentage of items evaluated with “yes” in the JBI checklists: ≥70% = high quality; 50–69% = moderate; <50% = low. Randomized controlled trial—RCT.

**Table A2:** Item-level risk-of-bias assessment across predefined domains.

Author, Year	Tool	Q1	Q2	Q3	Q4	Q5	Q6	Q7	Q8	Q9	Q10	Q11	Q12	Q13
Couto et al., 2019 [34]	Quasi-experimental	Y	Y	Y	N	Y	Y	Y	Y	Y	-	-	-	-
Ferreira-Brito et al., 2020 [29]	Cross-sectional	Y	Y	Y	Y	N	N	Y	Y	-	-	-	-	-
Gamito et al., 2020 [30]	Quasi-experimental	Y	Y	Y	Y	Y	Y	Y	Y	Y	-	-	-	-
Lopes et al., 2016 [33]	Quasi-experimental	Y	Y	Y	N	N	Y	Y	Y	Y	-	-	-	-
Oliveira et al., 2021 [31]	RCT	Y	U	Y	N	N	N	Y	Y	Y	Y	Y	Y	Y
Silva et al., 2025a [32]	RCT	Y	U	Y	N	U	U	Y	Y	Y	Y	Y	Y	Y
Silva et al., 2025b [35]	RCT	Y	U	Y	N	U	U	Y	Y	Y	Y	Y	Y	Y

Note. Y = Yes (criterion met); N = No (criterion not met); U = Unclear (insufficient information reported); “-” = Not applicable. All judgments were based on the methodological information explicitly reported in the original studies, following Joanna Briggs Institute (JBI) critical appraisal tools. When information was not reported, items were classified as “Unclear” or “No”, depending on the domain. As different study designs were included, not all items apply to all studies.
